# Adaptive bacterial response to low level chlorhexidine exposure and its implications for hand hygiene

**DOI:** 10.15698/mic2019.07.683

**Published:** 2019-03-07

**Authors:** Günter Kampf

**Affiliations:** 1 Institute for Hygiene and Environmental Medicine, University Medicine Greifswald, Ferdinand-Sauerbruch-Straβe, 17475 Greifswald, Germany.

**Keywords:** chlorhexidine digluconate, adaptation, resistance, cross-tolerance, low level exposure, MIC values

## Abstract

Chlorhexidine digluconate (CHG) is commonly used in healthcare, e.g. in skin antiseptics, antimicrobial soaps, alcohol-based hand rubs and oral or wound antiseptics. Aim of the literature review was to evaluate the potential of bacteria to adapt to low level CHG exposure. A maximum 4fold MIC increase to CHG was found after low level exposure in most of the 71 evaluated bacterial species. A strong adaptive mostly stable MIC change was described in strains or isolates of the healthcare-associated species *E. coli*, *S. marcescens* and *P. aeruginosa* (up to 500fold, 128fold or 32fold, respectively). The highest MIC values after adaptation were 2,048 mg/l (*S. marcescens*) and 1,024 mg/l (*P. aeruginosa*). A new resistance to tetracycline, gentamicin, meropeneme or triclosan was found in some adapted isolates. In *E. coli* horizontal gene transfer was induced (sulfonamide resistance by conjugation), pointing out an additional risk of sublethal CHG. The use of CHG in patient care - but also all other settings such as consumer products and households - should therefore be critically assessed and restricted to indications with a proven health benefit or justifiable public health benefits. Additional CHG has no health benefit when used in alcohol-based hand rubs and is not recommended by the WHO. For routine hand washing of soiled hands the use of plain soap is sufficient, CHG in soaps has no health benefit. In surgical hand antisepsis alcohol-based hand rubs should be preferred to CHG soaps. Implementation of these principles will help to reduce avoidable selection pressure.

## INTRODUCTION

Chlorhexidine digluconate (CHG) is a commonly used antiseptic agent in human healthcare and veterinary medicine, mainly used for hand hygiene (e.g. at 2% - 4% as the only active agent in antiseptic soaps or at 0.5% or 1% as an additional active agent in alcohol-based hand rubs), in alcohol-based skin antiseptics at 2% and in mouth rinse solutions at 0.12% - 0.2% [1]. The widespread CHG use in various types of applications has probably lead to an increase of acquired bacterial resistances, mainly in Gram-negative species such as *Pseudomonas aeruginosa* (minimal inhibitory concentration (MIC) of up to 800 mg/l), *Serratia marcescens* (MIC of up to 400 mg/l) or *Klebsiella pneumoniae* (MIC of up to 256 mg/l) [1]. In some types of applications such as skin antiseptics CHG has been shown to reduce healthcare associated infections, e.g. catheter-associated bloodstream infections [2]. Recent evidence also suggests a contribution to the prevention of surgical site infections [3] although the single effect of CHG for this application is still under controversial debate [4–6].

Its widespread use in hand hygiene by healthcare workers in many countries suggests to look specifically at all possible applications in this area. The WHO has published a recommendation on hand hygiene for healthcare in 2009 with the aim to reduce healthcare-associated infections [7]. Three types of applications can be distinguished. The use of alcohol-based hand rubs is recommended on clean hands in five specific clinical situations: before touching a patient, before clean or aseptic procedures, after body fluid exposure, after touching a patient and after touching patient surroundings [7, 8]. Hand washing with either plain soap or antiseptic soap and water is recommended for visibly soiled hands or in case of contamination with spore-forming bacteria such as *Clostridium difficile* [7]. The third type of application is in the surgical theater. Healthcare workers should decontaminate their hands prior to donning sterile surgical gloves with either alcohol-based hand rubs (surgical hand disinfection) or with antimicrobial soaps (surgical scrubbing) [7].

**Figure 1:**
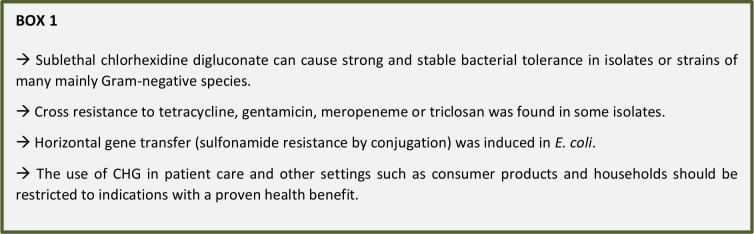


In the past years there is an increasing concern on the development of resistance not only to antibiotics but also to antiseptic agents which are essential to limit the spread of multidrug-resistant pathogens in healthcare [9, 10]. Some antiseptic agents are more likely than others to cause a bacterial tolerance or even resistance [11]. Aim of the review is therefore to evaluate the potential of CHG to cause an adaptive bacterial response during exposure to sublethal concentrations and to propose reasonable implications for the use of CHG in hand hygiene.

## RESULTS

### General remark

The magnitude of any adaptive response to CHG is expressed as an MIC change and assigned to one of the following three categories: No adaptive response (no MIC increase), weak adaptive response (MIC increase ≤ 4fold) and strong adaptive response (MIC increase > 4fold). For some bacterial species two or more studies were found resulting in data from various isolates or strains. That is why some bacterial species can be found in two or three categories depending on the results obtained with the various isolates or strains of the same species.

### Adaptive bacterial response in Gram-negative species

No adaptive response was found in isolates or strains of 15 species (*Acinetobacter baumannii*, *Aeromonas hydrophila*, *Campylobacter coli*, *Campylobacter jejuni*, *Chryseobacterium indologenes*, *Citrobacter* spp., *Cronobacter sakazakii*, *E. coli*, *K. pneumoniae*, *Moraxella osloensis*, *P. aeruginosa*, *Pseudomonas nitroreductans*, *Pseudomonas putida*, *Pseudoxanthomonas* spp. and *Sphingobacterium multivorum*). Some isolates or strains of 12 species were able to express a weak adaptive response (MIC increase ≤ 4fold) such as *A. xylosoxidans*, *A. jandaei*, *Chrysobacterium* spp., *E. cloacae*, *Enterobacter* spp., *E. coli*, *H. gallinarum*, *K. pneumoniae*, *P. aeruginosa*, *S. Typhimurium*, *Serratia* spp. and *S. maltophilia* (**Table 1**).

**TABLE 1:** Adaptive response of Gram-negative bacterial species to sublethal CHG exposure, adapted from [35].

Species	Strain / isolate	Type of exposure	Increase in MIC	MIC_max_ (mg/l)	Stability	Associated changes	Ref
*A. xylosoxidans*	Domestic drain biofilm isolate MBRG 4.31	14 d at various concentrations	2fold	31.2	No data	None reported	[36]
*A. baumannii*	Strain MBRG15.1 from a domestic kitchen drain biofilm	14 passages at various concentrations	None	7.8	Not applicable	None reported	[37]
*A. baylyi*	Strain ADP1	30 min at 0.000001%	Protection from lethal CHG concentration (0.00007%)	No data	No data	More resistance to a lethal hydrogen peroxide concentration (300 mM)	[38]
*A. hydrophila*	Domestic drain biofilm isolate MBRG 4.3	14 d at various concentrations	None	15.6	Not applicable	None reported	[36]
*A. jandaei*	Domestic drain biofilm isolate MBRG 9.11	14 d at various concentrations	2fold	15.6	No data	None reported	[36]
*A. proteolyticus*	Domestic drain biofilm isolate MBRG 9.12	14 d at various concentrations	16fold	125	No data	None reported	[36]
*B. fragilis*	ATCC 25285	12 h at 0.06%	No data	No data	Not applicable	Induction of multiple antibiotic resistance*; 2.7fold – 6fold increase of 6 efflux pumps	[39]
*B. cenocepacia*	6 strains from clinical and environmental habitats	Up to 28 d at 15 mg/l	Survival	100	No data	No degradation of CHG	[40]
*B. cepacia*	ATCC BAA-245	40 d at various concentrations	8fold	29	Unstable for 14 d	Decrease biofilm formation	[41]
*B. cepacia complex*	*B. lata* strain 383	5 min at 50 mg/l	No data	700	Not applicable	Reduced susceptibility** to ceftazidime (30 – 33 mm), ciprofloxacin (11 – 20 mm) and imipenem (15 – 21 mm; 2 of 4 experiments) and to meropenem (33 mm; 1 of 4 experiments); up-regulation of transporter and efflux pump genes	[42]
*C. coli*	ATCC 33559 and a poultry isolate	Up to 15 passages with gradually higher concentrations	None	0.031	Not applicable	None described	[15]
*C. jejuni*	NCTC 11168, ATCC 33560 and a poultry isolate	Up to 15 passages with gradually higher concentrations	None	1	Not applicable	None described	[15]
*C. indologenes*	MRBG 4.29 (kitchen drain biofilm isolate)	40 d at various concentrations	None	7.3	Not applicable	None described	[41]
*C. indologenes*	Domestic drain biofilm isolate MBRG 9.15	14 d at various concentrations	None	31.2	Not applicable	None reported	[36]
*Chrysobacterium* spp.	Domestic drain biofilm isolate MBRG 9.17	14 d at various concentrations	2fold	7.8	No data	None reported	[36]
*Chrysobacterium* spp.	2 biocide-sensitive strains from organic foods	Several passages with gradually higher concentrations	5fold – 6fold	30	Unstable	Cross-adaptation* to benzalkoniumchloride (2fold - 100fold; 2 strains), triclosan (4fold; 1 strain) and didecyldimethyl- ammonium bromide (16fold; 1 strain); cross-resistance* to cefotaxime and ceftazidime (2 strains each), sulfamethoxazole, ampicillin and tetracycline (1 strain each)	[43]
*Citrobacter* spp.	Domestic drain biofilm isolate MBRG 9.18	14 d at various concentrations	None	1.9	Not applicable	None reported	[36]
*C. sakazakii*	Strain MBRG15.5 from a domestic kitchen drain biofilm	14 passages at various concentrations	None	7.8	Not applicable	None reported	[37]
*E. cloacae*	2 biocide-sensitive strains from organic foods	Several passages with gradually higher concentrations	10fold – 16fold	80	Stable for 20 subcultures (1 strain)	Cross-adaptation* to benzalkoniumchloride (6fold; 2 strains), triclosan (6fold - 15fold; 2 strains) and didecyldimethylammonium bromide (6fold; 1 strain); cross-resistance* to imipenem, ceftazidime and sulfamethoxazole (2 strains each), cefotaxime and tetracycline (1 strain each)	[43]
*E. ludwigii*	2 biocide-sensitive strains from organic foods	Several passages with gradually higher concentrations	6fold – 8fold	40	Unstable	Cross-adaptation* to benzalkoniumchloride (6fold – 8fold; 2 strains), triclosan (8fold – 10fold; 2 strains) and didecyldimethylammonium bromide (4fold – 6fold; 2 strains); cross-resistance* to imipenem, ceftazidime and sulfamethoxazole (2 strains each)	[43]
*Enterobacter* spp.	6 biocide-sensitive strains from organic foods	Several passages with gradually higher concentrations	4fold – 10fold	80	Stable for 20 subcultures (1 strain)	Cross-adaptation* to benzalkoniumchloride (3fold – 20fold; 6 strains), triclosan (4fold – 100fold; 6 strains) and didecyldimethylammonium bromide (4fold – 6fold; 3 strains); cross-resistance* to ceftazidime and imipenem (3 strains each), cefotaxime and sulfamethoxazole (2 strains each)	[43]
*E. coli*	ATCC 25922	40 d at various concentrations	None	7.3	Not applicable	None described	[41]
*E. coli*	NCIMB 8879	6 x 48 h at variable concentrations	None	0.7	Not applicable	None reported	[44]
*E. coli*	ATCC 25922 and strain MBRG15.4 from a domestic kitchen drain biofilm	14 passages at various concentrations	1.5fold - 5fold	11.7	Stable for 14 d	None reported	[37]
*E. coli*	NCIMB 8545	0.00005% for 30 s, 5 min and 24 h	≤ 6fold	39	Unstable for 10 d	No increase of MBC; unstable resistance** to tobramycin	[45]
*E. coli*	NCTC 8196	12 w at various concentrations	32fold	No data	No data	None described	[46]
*E. coli*	NCTC 12900 strain O157	6 passages at variable concentrations	Approx. 500fold	Approx. 500	Stable for 30 d	Increased tolerance** to triclosan (15 mm)	[47]
*E. coli*	CV601	24.4 µg/l for 3 h	No data	4.9	Not applicable	Induction of horizontal gene transfer (sulfonamide resistance by conjugation)	[48]
*H. gallinarum*	Domestic drain biofilm isolate MBRG 4.27	14 d at various concentrations	2fold	31.2	No data	None reported	[36]
*K. oxytoca*	2 biocide-sensitive strains from organic foods	Several passages with gradually higher concentrations	2fold – 8fold	40	Unstable	Cross-adaptation* to benzalkoniumchloride (60fold; 1 strain), triclosan (3fold – 8fold; 2 strains) and didecyldimethyl- ammonium bromide (6fold; 1 strain)	[43]
*K. pneumoniae*	7 “Murray isolates” from the pre-CHG era	Up to 5 w at various concentrations	None (5 isolates)	4fold (2 isolates)	256	Stable for 10 d	None reported	[49]
*K. pneumoniae*	7 modern isolates / strains	Up to 5 w at various concentrations	4fold - 16fold	> 512	Stable for 10 d	None reported	[49]
*K. pneumoniae*	6 clinical strains with a variety of antibiotic resistance markers	6 passages of 2 days at various concentrations	4fold – 16fold	512	Stable for 10 d	Cross-resistance*** to colistin (6 strains); no cross-adaptation to benzalkoniumchloride, octenidine, hexadecylpyridinium chloride	monohydrate and ethanol	[16]
*K. pneumoniae*	ATCC 13883	40 d at various concentrations	6.9fold	14.5	Stable for 14 d	Increase biofilm formation	[41]
*Klebsiella* spp.	Biocide-sensitive strain from organic foods	Several passages with gradually higher concentrations	2fold	30	Unstable	Cross-adaptation* to benzalkoniumchloride (12fold) and triclosan (12fold); cross-resistance* to imipenem and ceftazidime	[43]
*M. osloensis*	Strain MBRG15.3 from a domestic kitchen drain biofilm	14 passages at various concentrations	None	2.0	Not applicable	None reported	[37]
*P. agglomerans*	5 biocide-sensitive strains from organic foods	Several passages with gradually higher concentrations	5fold – 10fold	50	Unstable	Cross-adaptation* to benzalkoniumchloride (30fold – 40fold; 5 strains), triclosan (8fold – 100fold; 5 strains) and didecyldimethylammonium bromide (4fold - 6fold; 2 strains); cross-resistance* to cefotaxime and ceftazidime (3 strains each), tetracycline and sulfamethoxazole (2 strains each) and imipenem (1 strain)	[43]
*P. ananatis*	2 biocide-sensitive strains from organic foods	Several passages with gradually higher concentrations	10fold – 50fold	50	Unstable	Cross-adaptation* to benzalkoniumchloride (20fold – 30fold; 2 strains), triclosan (60fold – 100fold; 2 strains) and didecyldimethylammonium bromide (6fold; 2 strains); cross-resistance* to cefotaxime (2 strains), sulfamethoxazole, imipenem, ceftazidime and tetracycline (1 strain each)	[43]
*Pantoea* spp.	3 biocide-sensitive strains from organic foods	Several passages with gradually higher concentrations	5fold – 16fold	80	Unstable	Cross-adaptation* to benzalkoniumchloride (6fold – 60fold; 2 strains), triclosan (8fold; 3 strains) and didecyldimethylammonium bromide (4fold - 6fold; 3 strains); cross-resistance* to tetracycline (2 strains), ampicillin, ceftazidime, cefotaxime, sulfamethoxazole and imipenem (1 strain each)	[43]
*P. aeruginosa*	178 CHG sensitive strains	Exposure to CHG	None	625	Not applicable	None reported	[50]
*P. aeruginosa*	ATCC 9027	40 d at various concentrations	2fold	14.5	Unstable for 14 d	None described	[41]
*P. aeruginosa*	ATCC 9027	14 passages at various concentrations	4fold	31.3	Stable for 14 d	None reported	[37]
*P. aeruginosa*	NCIMB 10421	6 x 48 h at variable concentrations	7fold	70	Stable for 15 d	High MICs to BAC did not change in a relevant extent	[44]
*P. aeruginosa*	NCTC 6749	12 w at various concentrations	8fold – 32fold	1,024	Stable for 7 w	None described	[46]
*P. nitroreductans*	Domestic drain biofilm isolate MBRG 4.6	14 d at various concentrations	None	3.9	Not applicable	None reported	[36]
*P. putida*	Strain MBRG15.2 from a domestic kitchen drain biofilm	14 passages at various concentrations	None	7.8	Not applicable	None reported	[37]
*Pseudomonas* spp.	Domestic drain biofilm isolate MBRG 9.14	14 d at various concentrations	16fold	15.6	No data	None reported	[36]
*Pseudoxanthomonas* spp.	Domestic drain biofilm isolate MBRG 9.20	14 d at various concentrations	None	0.97	Not applicable	None reported	[36]
*Ralstonia* spp.	Domestic drain biofilm isolate MBRG 4.13	14 d at various concentrations	21fold	167	No data	None reported	[36]
*S. Virchow*	Food isolate	6 passages at variable concentrations	Approx. 120fold	Approx. 120	Stable for 30 d	Increased tolerance** to triclosan (0 mm)	[47]
*Salmonella enterica serovar*	*Typhimurium*	Strain SL1344	5 min at 0.1, 0.5, 1 and 4 mg/l	13fold – 27fold	800	Unstable for 1 d	3fold – 67fold increase of tolerance*** to BAC	[51]
*Salmonella enterica serovar*	*Typhimurium*	Strain 14028S	5 min at 1 and 5 mg/l	3fold – 33fold	1,000	Unstable for 1 d	2.5fold – 20fold increase of tolerance*** to BAC	[51]
*S. enteritidis*	ATCC 13076	7 d of sublethal exposure	≥ 10fold	> 50	Unstable	None reported	[52]
*Salmonella* spp.	3 biocide-sensitive strains from organic foods	Several passages with gradually higher concentrations	5fold – 10fold	50	Unstable	Cross-adaptation* to benzalkoniumchloride (8fold – 30fold; 2 strains) and triclosan (4fold - 8fold; 3 strains) cross-resistance* to cefotaxime, nalidixic acid and imipenem (2 strains each), tetracycline and sulfamethoxazole (1 strain each)	[43]
*Salmonella* spp.	6 strains with higher MICs to biocidal products	8 days at increasing concentrations	50fold – 200fold (2 strains)	> 1,000	“stable”	One strain with increased tolerance***	to tetracycline (> 16 mg/l), chloramphenicol (8 mg/l) and nalidixic acid (16 mg/l)	[53]
*S. marcescens*	Strain GSU 86-828	7 d exposure to CHG-containing contact lens solutions	8fold	50	No data	Increased adherence to polyethylene	[54]
*S. marcescens*	ATCC 13880	40 d at various concentrations	9.6fold	116	Stable for 14 d	Increase biofilm formation	[41]
*S. marcescens*	Clinical isolate	12 w at various concentrations	32fold – 128fold	2,048	Stable for 7 w	None described	[46]
*Serratia* spp.	Not described	5 to 8 transfers	“resistance“ to CHG	No data	“stable”	None described	[55]
*S. multivorum*	Domestic drain biofilm isolate MBRG 9.19	14 d at various concentrations	None	15.6	Not applicable	None reported	[36]
*S. maltophilia*	Domestic drain biofilm isolate MBRG 9.13	14 d at various concentrations	4fold	62.5	No data	None reported	[36]
*S. maltophilia*	MRBG 4.17 (kitchen drain biofilm isolate)	40 d at various concentrations	6fold	29	Stable for 14 d	None described	[41]

* spiral gradient endpoint method;

** disc diffusion method;

*** broth microdilution;

**** macrodilution method

A strong but unstable MIC change (> 4fold) was found in isolates or strains of four species (*Burkholderia cepacia*, *E. coli*, *Salmonella enteritidis*, *Salmonella Typhimurium*). A strong and stable MIC change (> 4fold) was described for isolates or strains of seven species (*E. coli*, *K. pneumoniae*, *P. aeruginosa*, *Salmonella Virchow*, *Salmonella* spp., *S. marcescens*, *Stenotrophomonas maltophilia*). In isolates or strains of six species (*Acinetobacter baylyi*, *Acinetobacter proteolyticus*, *E. coli*, *Pseudomonas* spp., *Ralstonia* spp., *S. marcescens*) the adaptive response was strong but its stability was not described.

Selected strains or isolates revealed substantial MIC changes: *E. coli* (up to 500fold), *Salmonella* spp. (up to 200fold), *S. marcescens* (up to 128fold), *P. aeruginosa* (up to 32fold), or *A. proteolyticus*, *K. pneumoniae*, and *Pseudomonas* spp. (all up to 16fold). The highest MIC values after adaptation were found in *S. marcescens* (2,048 mg/l), *P. aeruginosa* (1,024 mg/l), *Salmonella* spp. (> 1,000 mg/l), *B. cepacia complex* (700 mg/l), *K. pneumoniae* (> 512 mg/l) and *E. coli* (500 mg/l). Most maximum MIC values are above the proposed epidemiological cut-off value of 16–64 mg/l to determine CHG resistance in Gram-negative bacterial species [12].

Cross resistance to various antibiotics such as tetracycline, gentamicin or meropeneme was found in some isolates of *Bacterioides fragilis*, *B. cepacia complex* and *Salmonella* spp.. In addition, a lower susceptibility to other biocidal agents was described for *E. coli* and *S. Virchow* to triclosan, for *A. baylyi* to hydrogen peroxide and for *S. Typhimurium* to benzalkonium chloride (BAC).

Other adaptive changes include a significant up-regulation of efflux pump genes in *B. fragilis* and *B. cepacia complex*. Horizontal gene transfer (sulfonamide resistance by conjugation) was induced in *E. coli*. VanA-type vancomycin resistance gene expression was increased vanA *Enterococcus faecium* (≥ 10fold increase of vanHAX encoding). Enhanced biofilm formation was described for *K. pneumoniae* and *S. marcescens*, adherence to poly-ethylene was increased in *S. marcescens*. Biofilm formation was decreased in *B. cepacia*.

### Adaptive bacterial response in Gram-positive species

No adaptive response was found in isolates or strains from 18 species (*Bacillus cereus*, *Corynebacterium xerosis*, *Enterococcus saccharolyticus*, *Eubacterium* spp., *Methylobacterium phyllosphaerae*, *Micrococcus luteus*, *Staphylococcus aureus*, *Staphylococcus capitis*, *Staphylococcus caprae*, *Staphylococcus cohnii*, *Staphylococcus epidermidis*, *Staphylococcus haemolyticus*, *Staphylococcus hominis*, *Staphylococcus kloosii*, *Staphylococcus lugdenensis*, *Staphylococcus saprophyticus*, *Staphylococcus warneri* and *Streptococcus mutans*).

Some isolates or strains of 12 species ware able to express a weak adaptive response (MIC increase ≤ 4fold) such as *B. cereus, Corynebacterium pseudogenitalum*, *Corynebacterium renale* group, *Enterococcus casseliflavus*, *Enterococcus faecalis*, *E. faecium*, *M. luteus*, *S. aureus*, *S. capitis*, *S. haemolyticus*, *S. lugdenensis* and *S. warneri*.

A strong but unstable MIC change (> 4fold) was found in isolates or strains of *E. faecalis*. A strong MIC change (> 4fold) was also described for isolates or strains of *S. aureus* which could be stable or of unknown stability.

The largest MIC increase was noticed in *S. aureus* (up to 16fold) and *E. faecalis* (up to 6.7fold) leading to MIC values as high as 24.2 mg/l in *E. faecalis* and 20 mg/l in *S. aureus* (**Table 2**). Some maximum MIC values are above the proposed epidemiological cut-off value (8 mg/l for *S. aureus*) and some below (64 mg/l for *E. faecalis*) to determine CHG resistance in Gram-positive bacterial species [12].

**TABLE 2:** Adaptive response of Gram-positive bacterial species to sublethal CHG exposure, adapted from [35].

Species	Strain / isolate	Type of exposure	Increase in MIC	MIC_max_ (mg/l)	Stability	Associated changes	Ref
*B. cereus*	MRBG 4.21 (kitchen drain biofilm isolate)	40 d at various concentrations	None	14.5	Not applicable	None described	[41]
*B. cereus*	Domestic drain biofilm isolate MBRG 4.21	14 d at various concentrations	None	1.9	Not applicable	None reported	[36]
*B. cereus*	4 biocide-sensitive strains from organic foods	Several passages with gradually higher concentrations	6fold – 16fold	80	Stable for 20 subcultures (1 strain)	Cross-adaptation* to benzalkoniumchloride (≥ 100fold; 3 strains), triclosan (4fold – 36fold; 3 strains) and didecyldimethylammonium bromide (6fold; 2 strains); cross-resistance* to imipenem (4 strains), sulfamethoxazole (2 strains), ampicillin and tetracycline (1 strain each)	[43]
*B. licheniformis*	2 biocide-sensitive strains from organic foods	Several passages with gradually higher concentrations	4fold – 10fold	50	Unstable	Cross-adaptation* to benzalkoniumchloride (40fold - 75fold; 2 strains) and triclosan (8fold; 1 strain); cross-resistance* to imipenem (2 strains), cefotaxime and tetracycline (1 strain each)	[43]
*B. subtilis*	2 strains and 3 derivates	2 h at 0.00005%	No data	No data	Not applicable	No increase of transfer of the mobile genetic element Tn916, a conjugative transposon	[56]
*Bacillus* spp.	4 biocide-sensitive strains from organic foods	Several passages with gradually higher concentrations	4fold – 8fold	40	Unstable	Cross-adaptation* to benzalkoniumchloride (15fold – 100fold; 4 strains), triclosan (8fold; 4 strains) and didecyldimethylammonium bromide (4fold - 6fold; 2 strains); cross-resistance* to imipenem and sulfamethoxazole (4 strains each), cefotaxime and ceftazidime (1 strain each)	[43]
*C. pseudogenitalum*	Human skin isolate MBRG 9.24	14 d at various concentrations	4fold	3.9	No data	None reported	[36]
*C. renale* group	Human skin isolate MBRG 9.13	14 d at various concentrations	4fold	31.2	No data	None reported	[36]
*C. xerosis*	WIBG 1.2 (wound isolate)	40 d at various concentrations	None	3.6	Not applicable	None described	[41]
*E. casseliflavus*	3 biocide-sensitive strains from organic foods	Several passages with gradually higher concentrations	8fold – 20fold	100	Stable for 20 subcultures (1 strain)	Cross-adaptation* to benzalkoniumchloride (30fold - 100fold; 4 strains), triclosan (> 100fold; 1 strain) and didecyldimethylammonium bromide (4fold - 6fold; 2 strains); cross-resistance* to imipenem (3 strains), cefotaxime and tetracycline (1 strain each)	[43]
*E. durans*	Biocide-sensitive strain from organic foods	Several passages with gradually higher concentrations	10fold	50	Unstable	Cross-adaptation* to benzalkoniumchloride (≥ 100fold), triclosan (10fold) and didecyldimethylammonium bromide (16fold); cross-resistance* to imipenem and ampicillin	[43]
*E. faecalis*	1 strain of unknown origin	14 passages at various concentrations	2fold	7.8	Stable for 14 d	None reported	[37]
*E. faecalis*	Strain SS497	10 passages at various concentrations	3.7fold	11	Significant increase of surface hydrophobicity	No data	[57]
*E. faecalis*	WIBG 1.1 (wound isolate)	40 d at various concentrations	6.7fold	24.2	Unstable for 14 d	None described	[41]
*E. faecalis*	Biocide-sensitive strain from organic foods	Several passages with gradually higher concentrations	10fold	50	Unstable	Cross-adaptation* to benzalkoniumchloride (80fold) and didecyldimethylammonium bromide (8fold); cross-resistance* to imipenem and ceftazidime	[43]
*E. faecium*	9 biocide-sensitive strains from organic foods	Several passages with gradually higher concentrations	2fold – 16fold	80	Stable for 20 subcultures (1 strain)	Cross-adaptation* to benzalkoniumchloride (10fold - 100fold; 9 strains), triclosan (4fold - 100fold; 6 strains) and didecyldimethylammonium bromide (4fold - 8fold; 7 strains); cross-resistance* to imipenem (9 strains), tetracycline (4 strains), ampicillin (2 strains) cefotaxime and ceftazidime (1 strain each)	[43]
*E. faecium*	VRE strain 410 (skin and soft tissue infection isolate)	21 d at various concentrations	4fold	19.6	No data	Subpolulation with reduced susceptibility* to daptomycin including significant alterations in membrane phospholipids	[58]
*E. faecium*	3 vanA VRE strains	15 min at MIC	No data	No data	Not applicable	≥ 10fold increase of vanHAX encoding VanA-type vancomycin resistance and of liaXYZ associated with reduced daptomycin susceptibility; vanA upregulation was not strain or species specific; VRE was more susceptible to vancomycin in the presence of subinhibitory chlorhexidine	[59]
*E. saccharolyticus*	Domestic drain biofilm isolate MBRG 9.16	14 d at various concentrations	None	1.9	Not applicable	None reported	[36]
*Enterococcus* spp.	6 biocide-sensitive strains from organic foods	Several passages with gradually higher concentrations	2fold – 10fold	50	Unstable	Cross-adaptation* to benzalkoniumchloride (30fold - 100fold; 6 strains), triclosan (4fold - 15fold; 5 strains) and didecyldimethylammonium bromide (4fold - 6fold; 4 strains); cross-resistance* to imipenem (6 strains), ceftazidime and sulfamethoxazole (5 strains each), cefotaxime (4 strains), tetracycline (3 strains) and ampicillin (2 strains)	[43]
*Eubacterium* spp.	Domestic drain biofilm isolate MBRG 4.14	14 d at various concentrations	None	31.2	Not applicable	None reported	[36]
*M. phyllosphaerae*	Domestic drain biofilm isolate MBRG 4.30	14 d at various concentrations	None	15.6	Not applicable	None reported	[36]
*M. luteus*	MRBG 9.25 (skin isolate)	40 d at various concentrations	None	3.6	Not applicable	None described	[41]
*S. aureus*	ATCC 6538	40 d at various concentrations	None	3.6	Not applicable	None described	[41]
*S. aureus*	ATCC 6538	100 d at various concentrations	None	0.6	Not applicable	None described	[60]
*S. aureus*	NCTC 6571 plus 2 MRSA strains	Several passages with gradually higher concentrations	1.3fold – 2fold	1	“unstable”	None described	[61]
*S. aureus*	NCIMB 9518	0.00005% for 30 s, 5 min and 24 h	2fold – 5fold	20	Stable for 10 d	No increase of MBC	[45]
*S. aureus*	ATCC 6538	7 d of sublethal exposure	2.5fold	2.5	Unstable for 10 d	None reported	[52]
*S. aureus*	3 clinical MRSA strains	10 passages at various concentrations	≤ 4fold	8	No data	No change of PHMB susceptibility**	[62]
*S. aureus*	ATCC 6538	14 passages at various concentrations	4fold	7.8	Unstable for 14 d	None reported	[37]
*S. aureus*	ATCC 25923 and 14 clinical isolates	14 d at various sublethal concentrations	4fold - 6fold (6 isolates)	6.3	No data	Increased tolerance* to ciprofloxacin (4fold - 64fold; 10 isolates), tetracycline (4fold - 512fold; all isolates), gentamicin (4fold - 512fold; 8 isolates), amikacin (16fold - 512fold; 11 isolates), cefepime (8fold - 64fold; 11 isolates) and meropeneme (8fold - 64fold; 9 isolates)	[63]
*S. aureus*	NCTC 4163	12 w at various concentrations	16fold	No data	No data	None described	[46]
*S. aureus*	Strain SAU3 carrying plasmid pWG613	10 min at 0.00005%	No data	No data	Not applicable	No significant reduction of plasmid transfer frequency	[64]
*S. capitis*	MRBG 9.34 (skin isolate)	40 d at various concentrations	1.7fold	6	Stable for 14 d	None described	[41]
*S. capitis*	Human skin isolate MBRG 9.34	14 d at various concentrations	None	7.8	Not applicable	None reported	[36]
*S. caprae*	MRBG 9.3 (skin isolate)	40 d at various concentrations	None	3.6	Not applicable	None described	[41]
*S. caprae*	Human skin isolate MBRG 9.30	14 d at various concentrations	None	7.8	No data	None reported	[36]
*S. cohnii*	Human skin isolate MBRG 9.31	14 d at various concentrations	None	3.9	Not applicable	None reported	[36]
*S. epidermidis*	MRBG 9.33 (skin isolate)	40 d at various concentrations	None	9.7	Not applicable	None described	[41]
*S. epidermidis*	Human skin isolate M 9.33	14 d at various concentrations	None	7.8	Not applicable	None reported	[36]
*S. epidermidis*	CIP53124	1 d at various concentrations	No data	No data	Not applicable	Significant increase of biofilm formation at various sublethal concentrations	[65]
*S. haemolyticus*	Human skin isolate MBRG 9.35	14 d at various concentrations	None	15.6	Not applicable	None reported	[36]
*S. haemolyticus*	MRBG9.35 (skin isolate)	40 d at various concentrations	2.1fold	3	Unstable for 14 d	None described	[41]
*S. hominis*	Human skin isolate MBRG 9.37	14 d at various concentrations	None	7.8	Not applicable	None reported	[36]
*S. kloosii*	Human skin isolate MBRG 9.37	14 d at various concentrations	None	7.8	Not applicable	None reported	[36]
*S. lugdunensis*	Human skin isolate MBRG 9.36	14 d at various concentrations	None	15.6	Not applicable	None reported	[36]
*S. lugdunensis*	MRBG 9.36 (skin isolate)	40 d at various concentrations	4fold	3.6	Stable for 14 d	None described	[41]
*S. saprophyticus*	Human skin isolate MBRG 9.29	14 d at various concentrations	None	3.9	Not applicable	None reported	[36]
*S. saprophyticus*	4 biocide-sensitive strains from organic foods	Several passages with gradually higher concentrations	2fold – 10fold	50	Unstable	Cross-adaptation* to benzalkoniumchloride (25fold - 100fold; 4 strains), triclosan (4fold - 8fold; 3 strains) and didecyldimethylammonium bromide (6fold - 12fold; 2 strains); cross-resistance* to ceftazidime (4 strains), imipenem, sulfamethoxazole and cefotaxime (2 strains each) and tetracycline (1 strain)	[43]
*S. warneri*	MRBG 9.27 (skin isolate)	40 d at various concentrations	None	29	Not applicable	None described	[41]
*S. warneri*	Human skin isolate MBRG 9.27	14 d at various concentrations	2fold	15.6	No data	None reported	[36]
*S. xylosus*	Biocide-sensitive strain from organic foods	Several passages with gradually higher concentrations	4fold	20	Unstable	Cross-adaptation* to benzalkoniumchloride (> 100fold), triclosan (8fold) and didecyldimethylammonium bromide (20fold); cross-resistance* to ceftazidime, imipenem, sulfamethoxazole, cefotaxime and tetracycline	[43]
*Staphylococcus* spp.	3 biocide-sensitive strains from organic foods	Several passages with gradually higher concentrations	4fold – 10fold	50	Unstable	Cross-adaptation* to benzalkoniumchloride (4fold - 10fold; 3 strains), triclosan (8fold - 100fold; 3 strains) and didecyldimethylammonium bromide (6fold - 20fold; 3 strains); cross-resistance* to ceftazidime (1 strain)	[43]
*S. mutans*	Strain UA159	10 passages at various concentrations	None	3	Not applicable	None reported	[57]

* broth microdilution;

** macrodilution method

Cross tolerance to various antibiotics such as tetracycline, gentamicin or meropeneme could be found in some isolates of *S. aureus*. In *E. faecium* (vancomycin-resistant enterococcus; VRE) a more than 10fold vanA up-regulation was detected as well as reduced daptomycin susceptibility. An increase in biofilm formation was described in *S. epidermidis*.

## DISCUSSION

The strongest adaptation to low level CHG exposure was found in common nosocomial pathogens such as *E. coli* (up to 500fold MIC increase), *S. marcescens* (up to 128fold MIC increase), *P. aeruginosa* (up to 32fold MIC increase) and *K. pneumoniae* (up to 16fold MIC increase). After sublethal exposure the highest MIC values were also found in common nosocomial pathogens such as *S. marcescens* (2,048 mg/l), *P. aeruginosa* (1,024 mg/l), *K. pneumoniae* (> 512 mg/l) and *E. coli* (500 mg/l), It is probably no coincidence that these pathogens are among those species considered to have extreme or even pan resistance to antibiotics [13].

Low level CHG exposure also reduced the susceptibility to selected antibiotics in *Burkholderia* spp. or *Salmonella* spp. In *Burkholderia* spp. an up-regulation of transporter and efflux pump genes was found. Efflux pumps are often not agent-specific and may well result in resistance to other biocidal agents or antibiotics [1]. A quite alarming finding was that horizontal gene transfer was induced in *E. coli* by low level CHG exposure enabling the faster spread of resistance genes within the bacterial community.

Some mechanisms of the adaptive response have been described. Increased expression of efflux pumps is recognized as a mechanism of antibiotic and biocide resistance. The pumps may have limited or broad substrates, the so-called multiple drug resistance pumps [14]. The multiple antibiotic resistance (mar) locus and mar regulon in *E. coli* and other members of the enterobacteriaceae is a paradigm for a generalized response locus leading to increased expression of efflux pumps. One such pump, the AcrAB pump, extrudes biocides such as triclosan, chlorhexidine and quaternary ammonium compounds as well as multiple antibiotics [14]. In *P. aeruginosa*, a number of multidrug efflux pumps export a broad range of substrates [14]. In *C. jejuni* and *C. coli* active efflux was identified in adapted strains. In addition, the outer membrane protein profiles had changed, along with morphological changes [15]. In *K. pneumoniae* CHG adaptation was associated with mutations in the two-component regulator phoPQ and a putative Tet repressor gene (smvR) adjacent to the major facilitator superfamily (MFS) efflux pump gene, smvA [16]. And in *Salmonella* spp. a defense network was described that involved multiple cell targets including those associated with the synthesis and modification of the cell wall, the SOS response, virulence, and a shift in cellular metabolism toward anoxic pathways. In addition, results indicated that CHG tolerance was associated with more extensive modifications of the same cellular processes involved in this proposed network, as well as a divergent defense response involving the up-regulation of additional targets such as the flagellar apparatus and an altered cellular phosphate metabolism [17].

A major limitation of this review is that most of the data were obtained in laboratories under defined conditions. The findings are certainly suitable to describe the potential for adaptation to CHG. But it is less clear if or how the findings are transferred to the clinic. In 2002 Block *et al.* described that the MIC for CHG was higher among clinical isolates when more CHG was used for any type of application [18]. A similar correlation between CHG usage and MIC values was described in 2018 with *S. aureus* [19]. Lindford *et al.* described an outbreak by MDR *A. baumannii* in a burn unit. One of the measures to finally control the outbreak was to reduce moist low-concentration CHG dressings on burn wounds [20]. And yet the clinical impact of an elevated MIC value remains under controversial debate [21]. In hand hygiene it is known that a low bactericidal effect of CHG on the skin can only be achieved in the presence of small volumes of water, the water released by the skin as transepidermal water loss does not seem to be sufficient [22]. If the water realised by the skin is sufficient to allow adaptive changes of the bacterial species on the skin is currently not know. And yet, the triclosan tale strongly suggested that “a chemical that constantly stresses bacteria to adapt, and behaviour that promotes antibiotic resistance needs to be stopped immediately when the benefits are null” [10]. CHG is obviously such a chemical that constantly stresses bacteria to adapt. Even if the clinical impact of isolates or strains with elevated MIC values cannot finally be evaluated at the moment it seems justified restricting the use of CHG to applications where health benefits are associated with its use.

## IMPLICATIONS FOR HAND HYGIENE

### Alcohol-based hand rubs

In alcohol-based hand rubs with additional CHG used for hygienic hand disinfection there is no sound evidence for an additional effect of CHG *in vitro* [23]. There is also no evidence on the prevention of any type of healthcare-associated infection by the additional CHG in hand rubs. But there are obvious risks such as acquired bacterial resistance, anaphylactic reactions or skin irritation [24]. Its use in the immediate patient environment may therefore contribute to the selection pressure especially when the CHG concentration is sublethal [20]. Additional biocidal agents in alcohol-based hand rubs such as CHG are not recommended by the WHO [7].

The same applies to hand rubs used for surgical hand disinfection [24]. For surgical hand disinfection additional biocidal agents such as CHG are not recommended because they do not contribute to the prevention of surgical site infections [3, 25]. Replacing hand rubs with additional CHG by hand rubs without CHG will help to reduce avoidable CHG selection pressure. They should, however, have an equivalent efficacy, dermal tolerance and user acceptability [26].

### Antimicrobial soaps

Another simple option to reduce CHG selection pressure is to ban CHG soaps in healthcare for regular hand washing. Based on the WHO recommendation for hand hygiene from 2009 hand washing is recommended to wash hands when they are visibly soiled. The use of plain soap, however, is adequate, there is no health benefit for antimicrobial soaps [7].

Another possible use of antimicrobial soaps is prior to surgery. Surgical scrubbing usually lasts for 6–10 min of scrubbing time and consumes between 5 and 20 l water per scrub [27–29]. Surgical scrub products may only be effective with additional post-scrub water-based CHG treatments of the hands which pose an additional contamination and selection pressure risk [30, 31]. Alcohol-based hand rubs with an appropriate concentration of alcohol(s) have a stronger effect on the resident hand flora, require typically 1.5 min for application, cause less skin irritation [32] and do not pose any selection pressure to bacterial species due to their volatility [33, 34].

## CONCLUSION

Overall, the evidence on the adaptive potential of various pathogens to low level CHG exposure strongly suggests to critically review the use of CHG in patient care and to eliminate it in all applications where no health benefit has been shown or is realistically expectable.

## METHODS

A systematic literature search was conducted via the National Library of Medicine (PubMed) and via ScienceDirect (only research articles) on 10^th^ March 2018 and up-dated on 25^th^ June 2018 using the term chlorhexidine in combination with low level exposure (17 hits PubMed, 5 hits ScienceDirect), adaptive response (6/24), sublethal (27/72), resistance and MIC (142/640), and resistant and MIC (116/648). In addition, studies deemed suitable for this review were also included. Publications were included and results were extracted from them when they provided original data on any type of adaptive response to the exposure of bacteria to sublethal concentrations of CHG, corresponding changes of MICs (CHG, antibiotics, and other biocidal agents), survival in CHG solutions, efflux pump activity, gene expression or biofilm formation. Articles were excluded when they described only data on fungi, outbreaks, pseudo-outbreaks or infections caused by contaminated CHG products or solutions, only biochemical changes, an adaptive effect with other chlorhexidine salts or when a CHG solution or product was used for disinfection during an outbreak but without being the suspected or proven source. Reviews were also excluded and screened for any original information within the scope of the review.

The susceptibility of isolates or strains to CHG is described as the minimum inhibitory concentration (MIC value). In most studies it was described as a single value and is presented as such unless stated otherwise. The magnitude of any adaptive response to CHG is expressed as an MIC change and assigned to one of the following three categories: no adaptive response (no MIC increase), weak adaptive response (MIC increase ≤ 4fold) and strong adaptive response (MIC increase > 4fold).

## SUPPLEMENTAL MATERIAL

Click here for supplemental data file.

All supplemental data for this article are available online at http://www.microbialcell.com/researcharticles/2019a-kampf-microbial-cell/.

## Abbreviations:

BAC: – benzalkonium chloride,
CHG: – chlorhexidine digluconate,
MIC: – minimal inhibitory concentration,
PHMB: – polyhexanide.
