# Supplementary material for: Adaptive bacterial response to low level chlorhexidine exposure and its implications for hand hygiene
Source: Microb Cell. 2019 Mar 7;6(7):307–20. doi: 10.15698/mic2019.07.683 (PMC6600115; doi:10.15698/mic2019.07.683)
Supplement: Supplementary file 1 [file mic-06-307-s01.pdf]

Checklist: Hand hygiene in healthcare

Hygienic hand disinfection

- ☐ It should be performed in 5 specific clinical situations: before touching a patient, before clean or aseptic procedures, after body fluid exposure, after touching a patient and after touching patient surroundings.
- ☐ A sufficient volume of the hand rub should be applied to keep hands wet for the recommended application time (typically 30 s).
- ☐ Both hands should be completely covered with the hand rub.
- ☐ CHG has no health benefit in alcohol-based hand rubs and is not recommended by the WHO as an additional biocidal agent for hygienic hand disinfection.

Hand washing

- ☐ Hand washing with either plain soap or antiseptic soap and water is recommended for visibly soiled hands or in case of contamination with spore-forming bacteria such as *C. difficile*.
- ☐ For routine hand washing of soiled hands or hands contaminated with bacterial spores CHG in liquid soaps has no additional health benefit, plain soap is sufficient.

Surgical hand antisepsis

- ☐ It should be performed prior to donning sterile surgical gloves with either alcohol-based hand rubs (surgical hand disinfection) or with antimicrobial soaps (surgical scrubbing).
- ☐ Hand rubs based on an effective concentration of alcohol(s) should be preferred to CHG soaps because they are often more effective and less irritant to the skin.
- ☐ In alcohol-based hand rubs CHG has no health benefit and is not recommended by the WHO as an additional biocidal agent for surgical hand disinfection.

This checklist represents the Supplemental Material of the publication „Adaptive bacterial response to low level chlorhexidine exposure and its implications for hand hygiene“ by Kampf (2019)

Please cite as: Günter Kampf (**2019**). Adaptive bacterial response to low level chlorhexidine exposure and its implications for hand hygiene. **Microbial Cell** 6(7): 307-320. doi: 10.15698/mic2019.07.683
